# Life’s Simple 7 and ischemic heart disease in the general Australian population

**DOI:** 10.1371/journal.pone.0187020

**Published:** 2017-10-26

**Authors:** Yang Peng, Zhiqiang Wang, Bin Dong, Sifan Cao, Jie Hu, Odewumi Adegbija

**Affiliations:** 1 Centre for Chronic Disease, Centre for Clinical Research, The University of Queensland, Herston, Australia; 2 Institute of Child and Adolescent Health, School of Public Health, Peking University Health Science Center, Beijing, China; 3 Centre for Longitudinal and Life Course Research, School of Public Health, The University of Queensland, Herston, Australia; University of Bristol, UNITED KINGDOM

## Abstract

**Background:**

The American Heart Association released 7 modifiable factors, Life’s Simple 7, that are expected to improve cardiovascular health (CVH), but their contributions to ischemic heart disease (IHD) in the general Australians are not well clarified.

**Methods:**

We performed a cross-sectional study based on 7499 adults (≥18 years) who have tested for total cholesterol and fasting plasma glucose as part of 2011–12 Australian Health Survey. Poisson regression analyses were used to estimate the incidence rate ratios and population attributable fractions of those factors to IHD prevalence. Participants were classified into three CVH groups based on the number of ideal metrics: inadequate (0–2), average (3–4), and optimal (5–7). Logistic regression analyses were performed to elucidate the relationship between overall CVH and IHD prevalence.

**Results:**

357 participants were self-reported having IHD condition, with a weighted prevalence of 3.3%. Physical inactivity, elevated body mass index (BMI) and total cholesterol (TC) were independently associated with IHD. Compared to the inadequate category, participants in the optimal and average categories have a 78% [adjusted odds ratio (OR), 0.22; 95% confidence interval (CI), 0.03–1.96] and a 45% (adjusted OR, 0.55; 95% CI, 0.39–0.77) lower IHD risk. One more optimal metric was associated with an 18% lower IHD risk (adjusted OR, 0.82; 95% CI, 0.71–0.93).

**Conclusions:**

Our findings indicate that physical inactivity, raised BMI and elevated TC were independent modifiable risk factors of IHD in the general Australian population. The improvement of overall CVH may also reduce IHD risk among the general Australian adults.

## Introduction

Ischemic heart disease (IHD), one of the most popular cardiovascular diseases (CVDs), accounted for 13.3% of global all-causes of death in 2010 and the proportion has increased by more than one third compared to that in 1990 [1]. IHD ranked first in causes of both death and years of life lost (YLLs) worldwide [2]. In Australia, IHD is also one of the leading contributors of mortality and YLLs. It was reported that 17.9% of all-cause mortality was due to IHD among Australians [3]. According to the 2013 Global Burden of Disease Study, IHD was the leading cause of YLLs in Australia [4].

To measure and improve the cardiovascular health (CVH) in the general American population, the American Heart Association (AHA) recommended seven modifiable factors, also called Life’s Simple 7, namely are smoking status, body mass index (BMI), physical activity, dietary pattern, total cholesterol (TC), blood pressure, and fasting plasma glucose (FPG) [5]. There are a number of studies that have examined the individual and combined relationships between Life’s Simple 7 and CVD prevalence, incidence and/or mortality in the United States [6–8], Finland [9], China [10], and Korea [11]. The associations were still not well examined in the general Australian adults from a large national survey.

Our current study was based on an Australian representative sample, the Australian Health Survey (AHS), aiming to clarify the separate and combined associations between Life’s Simple 7 and IHD prevalence.

## Subjects and methods

### Study design and subjects

We used data from the core sample of the 2011–12 AHS [12], a national wide and population-based survey consisting of three arms: a general health survey, a nutrition and physical activity survey, and a voluntary biomedical survey which included participants from the first two arms. The survey was conducted using a stratified multistage sample that is representative of the general Australian population. Households that were living in very remote areas of Australia and discrete Aboriginal and Torres Strait Islander communities were not in scope. The core sample consisted of 24910 adults (≥ 18 years old). We restricted our study to those with TC and FPG (n = 7499). All participants provided written informed consent and our study was approved by The School of Medicine Low Risk Ethical Review Committee in the University of Queensland (approval number 2016-SOMILRE-0161).

### Life’s Simple 7

Individual modifiable factors were categorized as ideal and unideal, respectively. For smoking status, the participants were categorized into current/former smokers (unideal) and never smokers (ideal). For BMI status, participants were classified into ideal category if they had BMI values less than 25 kg/m^2^. Participants were regarded as having an ideal physical activity status if they had taken 150 minutes moderate, 75 minutes vigorous or 150 minutes combined moderate and vigorous physical activity last week. We included two dietary factors, usual daily intake of fruits and vegetables, in our analyses. Participants were categorized as having an ideal dietary pattern if they had sufficient fruits and vegetables intake, which was determined by 2013 Australian Dietary Guidelines [13]. Participants were considered to have an ideal TC status if they had TC concentration < 200 mg/dL and were not taking cholesterol-lowing medication. Participants were considered to have an ideal blood pressure status if they had systolic blood pressure <120 mmHg and diastolic blood pressure <80 mm Hg. For FPG, a value less than 100 mg/dL was considered as ideal status. We used the number of ideal metrics to measure the overall CVH and participants were divided into three groups (0–2, 3–4 and 5–7 ideal metrics), accordingly [14].

### Outcome measurement

The self-reported IHD prevalence was based on International Classification of Diseases -10, codes I20-I25. To be more specific, respondents were regarded as having an IHD condition if they had been told by a doctor or nurse that they had IHD and they currently have IHD while taking the survey.

### Covariates

The following variables were adjusted as covariates in our study: age, sex, education attainment, income status, and residence region. Education attainment was categorized as high (≥ 12 school years) and low (< 12 school years). Income status was evaluated by household income and dichotomized as low (≤ 50th percentile equivalised weekly household income) and high (> 50th percentile equivalised weekly household income). Residence region was classified into major cities, inner regional areas and other areas (outer regional and remote). They were included in the multivariate models along with the individual metric, number of optimal metrics or CVH categories.

### Statistical analysis

Firstly, we applied univariate and multivariate Poisson regression analysis to obtain the crude and adjusted incidence rate ratios (IRRs) and corresponding 95% confidence intervals (CIs) and they were used to measure the associations between modifiable factors and IHD occurrence.

Secondly, we calculated adjusted population attributable fractions (PAFs) based on the following equation to quantify the effects of each component on IHD reduction [15]. *Pe* is the prevalence of exposure and Rate Ratios (RRs) were replaced with adjusted IRR. Participants with available CVH metrics were included in the first and second analyses for specific metrics.

$$PAF=\frac{Pe\times (RR-1)}{1+Pe\times (RR-1)}$$

Thirdly, we calculated odds ratios (ORs) and 95% CIs using logistic regression analyses to explore the relationship between overall CVH and IHD risk. We treated the number of ideal metrics as a continuous and a categorical variable, respectively. Participants with missing values in one or more of Life’s Simple 7 components were not included in the analyses.

Biomedical weights were applied, using Jackknife method, as recommended by the Australian Bureau of Statistics (ABS) [16] to representative the in-scope population. All analyses used expanded confidentialised unit record files of the AHS core sample and were conducted within the ABS’s Remote Access Data Laboratory with Stata, version 10.0. A two-sided *P* value < 0.05 was considered statistically significant.

## Results

Among the 7499 eligible participants, 357 were positive for IHD occurrence and the weighted prevalence was 3.3%. For the seven metrics, FPG had the highest weighted ideal prevalence (83.6%), followed by smoking status (55.6%), blood pressure (44.2%), BMI (39.2%), TC (45.5%), physical activity (26.7%) and dietary pattern (4.8%). The details of metrics and covariates are summarized in Table 1.

**Table 1:** Distribution of IHD cases, stratified by the metrics and covariates.

Variables	Status	IHD, n (%) ^a^	Non-IHD, n (%) ^a^	*P* ^b^
Smoking	Ideal	132 (2.5)	3630 (97.5)	<0.01
	Unideal	225 (4.3)	3512 (95.7)	
BMI	Ideal	71 (1.9)	2324 (98.1)	<0.01
	Unideal	262 (4.1)	4470 (95.9)	
Physical activity	Ideal	35 (1.0)	1739 (99.0)	<0.01
	Unideal	322 (4.1)	5399 (95.9)	
Dietary pattern	Ideal	22 (3.6)	412 (96.4)	0.72
	Unideal	335 (3.3)	6730 (96.7)	
TC	Ideal	64 (1.5)	2763 (98.5)	<0.01
	Unideal	293 (4.8)	4379 (95.2)	
Blood pressure	Ideal	96 (1.9)	2732 (98.1)	<0.01
	Unideal	246 (4.5)	4147 (95.5)	
FPG	Ideal	214 (2.5)	5725 (97.5)	<0.01
	Unideal	143 (7.3)	1417 (92.7)	
Age	< 60 years	72 (1.1)	4751 (98.9)	<0.01
	≥ 60 years	285 (10.2)	2391 (89.8)	
Sex	Male	214 (4.2)	3115 (95.8)	<0.01
	Female	143 (2.5)	4027 (97.5)	
Education level	High	80 (1.6)	3653 (98.4)	<0.01
	Low	277 (5.7)	3489 (94.4)	
Income	High	73 (1.6)	3327 (98.4)	<0.01
	Low	257 (5.8)	3160 (94.2)	
Region	Major cities	178 (2.6)	4384 (97.4)	<0.01
	Inner regional	114 (5.1)	1595 (94.9)	
	Other	65 (5.4)	1163 (74.6)	

^a^ Numbers and percentages were presented as unweighted and weighted, respectively.

^b^ *P* values were from weighted chi-square tests.

BMI, body mass index; TC, total cholesterol; FPG, fasting plasma glucose.

**Fig 1:**
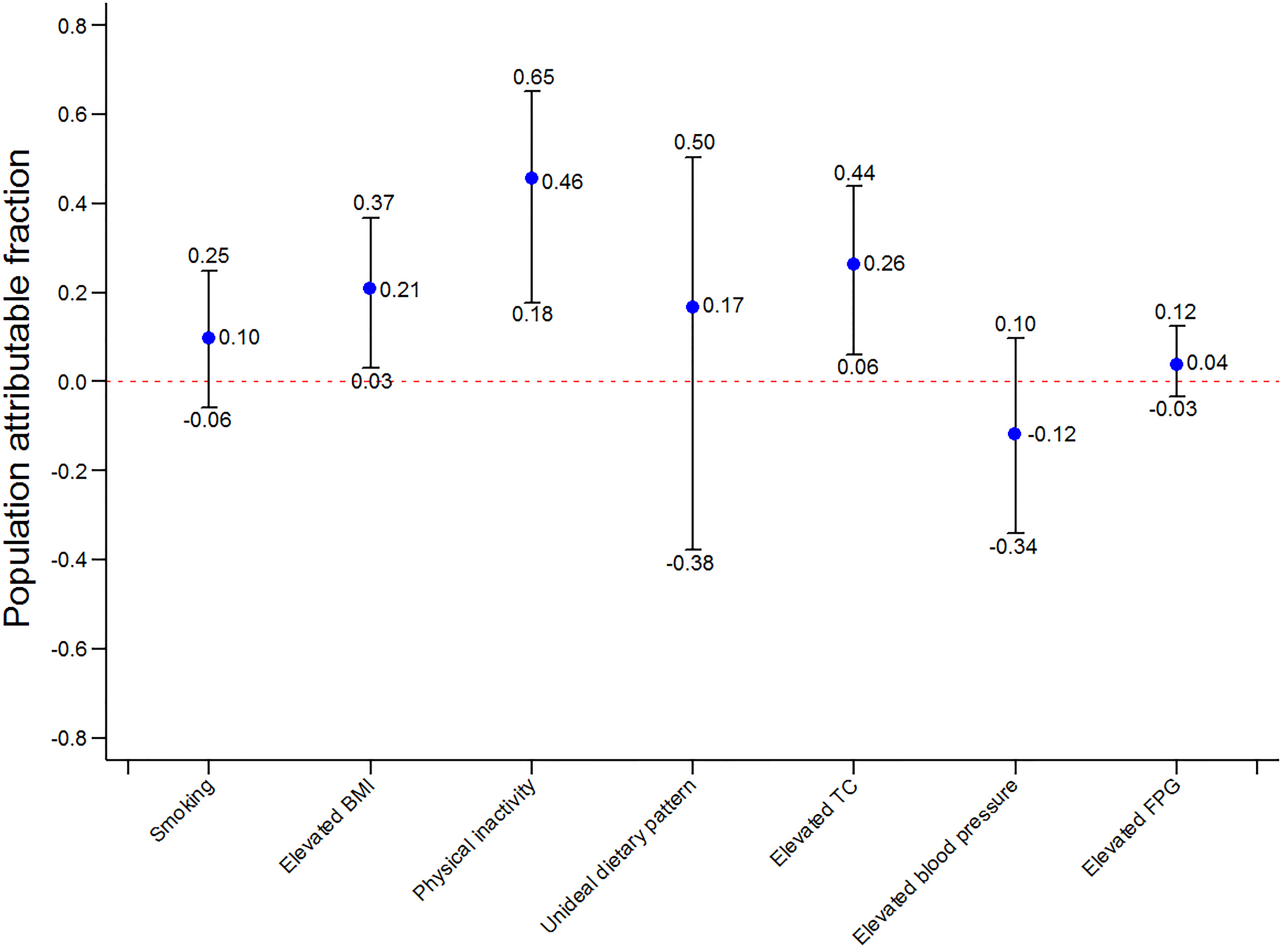
Adjusted population attributable fractions of each Life’s Simple 7 metric to ischemic heart disease.

In the univariate analysis, all the seven components, except for dietary pattern, were positively associated with increased IHD prevalence. After adjusted for confounders, physical inactivity (adjusted IRR: 2.10; 95% CI: 1.28–3.45, *P*<0.01), unideal TC (adjusted IRR: 1.58; 95% CI: 1.10–2.25, *P* = 0.01) and elevated BMI (adjusted IRR: 1.40; 1.05–1.87, *P* = 0.02) were still significantly associated with raised IHD risk (Table 2). We calculated adjusted PAFs to quantify the relative contributions of target influencing factors to IHD prevalence (Fig 1). Insufficient physical activity was the largest contributor to IHD prevalence (adjusted PAF: 46%; 95% CI: 0.18–0.65), followed by elevated TC (adjusted PAF: 26%; 95% CI: 0.06–0.44), and elevated BMI (adjusted PAF: 21%; 95% CI: 0.03–0.37).

**Table 2:** Individual associations between Life’s Simple 7 and IHD occurrence.

Variables	Crude IRR (95% CI)	*P*	Adjusted^a^ IRR (95% CI)	*P*
Smoking	1.70 (1.25–2.30)	<0.01	1.22 (0.89–1.67)	0.22
Elevated BMI	2.16 (1.58–2.94)	<0.01	1.40 (1.05–1.87)	0.02
Physical inactivity	3.92 (2.39–6.45)	<0.01	2.10 (1.28–3.45)	<0.01
Unideal dietary pattern	0.90 (0.50–1.62)	0.73	1.21 (0.71–2.07)	0.48
Elevated TC	3.25 (2.28–4.64)	<0.01	1.58 (1.10–2.25)	0.01
Elevated blood pressure	2.33 (1.61–3.39)	<0.01	0.83 (0.58–1.18)	0.29
Elevated FPG	2.86 (2.13–3.84)	<0.01	1.19 (0.84–1.68)	0.32

^a^ Adjusted for age, sex, education attainment, income, and residence region.

BMI, body mass index; TC, total cholesterol; FPG, fasting plasma glucose.

Table 3 shows the relationship between Life’s Simple 7 overall categories and IHD prevalence. Compared to those in the inadequate category, those in the optimal category had a 78% lower risk of IHD (adjusted OR: 0.22; 95% CI: 0.03–1.96) and those in the average category had a 45% lower risk (adjusted OR: 0.55; 95% CI: 0.39–0.77). On average, one more ideal metric was associated with an 18% lower risk of IHD (adjusted OR: 0.82; 95% CI: 0.71–0.93).

**Table 3:** Number of ideal metrics and IHD presence.

Ideal metrics number	IHD cases/participants	Crude OR (95% CI)	*P*	Adjusted ^a^ OR (95% CI)	*P*
0–2	241/3342	referent	—	referent	—
3–4	73/2786	0.28 (0.19–0.41)	<0.01	0.55 (0.39–0.77)	<0.01
5–7	4/874	0.03 (0.00–0.23)	<0.01	0.22 (0.03–1.96)	0.17
One more ideal metric	—	0.56 (0.50–0.63)	<0.01	0.82 (0.71–0.93)	<0.01

^a^ Adjusted for age, sex, education attainment, income, and residence region.

IHD, ischemic heart disease; OR, odds ratio.

## Discussion

To the best of our knowledge, it is the first study that explored the individual and combined effects of Life’s Simple 7 on IHD prevalence among the general Australians. We observed that physical inactivity, elevated BMI and raised TC are independent risk factors of IHD prevalence. The higher optimal metric number was associated with reduced IHD risk.

Physical inactivity was the most significant contributor to IHD in our study, with adjusted PAF of 46%. A recent meta-analysis revealed that, compared with insufficiently active participants (reporting less than 600 MET minutes/week of total physical activity), the risk of IHD among those in the low active (600–3999 MET minutes/week), moderately active (4000–7999 MET minutes/week), and highly active (≥8000 MET minutes/week) categories has reduced by 16%, 23%, and 25%, respectively [17]. In our study, less than 1 out of 4 participants met the requirement of physical activity. Thus, the policy makers should pay greater attention to the physical activity promotion in the general Australians.

Raised BMI was observed as a significant influencing factor of IHD, which is in agreement with several studies. A large-scale collaborative analysis identified one standard deviation (4.56 kg/m^2^) increase in BMI was independently associated with 11% higher risk of coronary heart disease incidence [18]. Another collaborative study demonstrated the positive relationship between BMI and IHD mortality [19]. It observed that, in the upper range of BMI (25–50 kg/m^2^), each 5 kg/m^2^ higher BMI was associated with a 39% higher IHD mortality.

We also found the independent and positive relationship between TC and IHD risk. A recent meta-analysis of more than one million persons from 97 prospective studies indicated that each one mmol/L increase in TC raised the risk of coronary heart disease incidence by 24% and 20% in male and female, which is consistent with our findings [20]. While, a recent study, using National Health and Nutrition Examination Survey data, found a non-significant relationship between TC and IHD mortality [7]. Given the conflicting findings, more researches on the TC-IHD relationship are warranted.

Smoking, elevated blood pressure, and raised FPG were significant IHD contributors in the unadjusted analysis. However, the associations were attenuated after adjustment for several socio-economic factors (Table 2). The potential role of those socio-economic variables on CVD risk was indicated by several previous studies [21–24]. Also, the IHD prevalence varied by the status of those factors in the current study (Table 1). We failed to detect the role of dietary pattern in both univariate and multivariate models, which is in agreement with several studies [14, 25, 26]. While, others displayed its role in CVD risk reduction [10, 27]. The different measurements of dietary pattern may partially explain the conflicting findings.

We have found that the higher number of ideal metrics was greatly related to IHD risk reduction. Our findings are consistent with several previous studies, which also found the inverse relationship between overall CVH and CVD risk [28, 29]. Our results suggested that some components may not individually relate to IHD risk, but they are likely to interrelate with other Life’s Simple 7 metrics and have synergistic effects on IHD risk.

Our study has some limitations. First, it is a cross-sectional study and we are unable to examine the temporality between influencing factors and IHD incidence or mortality. Second, we used modified metric definitions compared to those outlined by the AHA [5] due to dataset structures, and the modifications may make our results incomparable to other studies. Thirdly, we did not compare the IHD risk differences for those in the same CVH groups giving the limited sample size. In addition, some variables, like dietary pattern, smoking status and the IHD status, were self-reported and thus may brought about misclassifications.

## Conclusions

In summary, we observed physical inactivity, raised BMI and TC as significant risk factors and contributors of IHD presence in the general Australian adults. The higher number of optimal Life’s Simple 7 metrics was associated with lower risk of IHD.
